# Efficient Production of High-Quality Infrared-Assisted Spouted Bed-Dried *Areca taro* Based on the Drying Temperature and Cutting Size Control

**DOI:** 10.3390/foods13020260

**Published:** 2024-01-14

**Authors:** Yitong Zhao, Fanqianhui Yu

**Affiliations:** 1 Haide College, Ocean University of China, Qingdao 266100, China; zyt2361246926@163.com; 2 Department of Computer Science and Technology, Ocean University of China, Qingdao 266100, China; 3 College of Food Science and Engineering, Ocean University of China, Qingdao 266003, China

**Keywords:** *Areca taro*, infrared-assisted spouted bed drying technology, antioxidant activity, optimal parameters, energy consumption

## Abstract

The purpose of this study was to apply infrared-assisted spouted bed drying (IRSBD) technology for *Areca taro* drying and to investigate the effects of different parameters on its drying quality. Specifically, in order to determine the suitable conditions for IRSBD, the effects of different drying temperatures (45 °C, 50 °C, 55 °C, and 60 °C) and cutting sizes (6 × 6 × 6 mm, 8 × 8 × 8 mm, 10 × 10 × 10 mm, and 12 × 12 × 12 mm) on the drying characteristics, temperature uniformity, and quality properties (including colour, rehydration ratio, total phenol content, total flavonoid content, and antioxidant activity) of *Areca taro* were studied. The results showed that the optimal drying condition was the sample with a cutting size of 10 × 10 × 10 mm and drying at 50 °C, which yielded the dried sample with the best colour, highest total phenol and flavonoid contents, maximum antioxidant capacity, and rehydration ratio.

## 1. Introduction

*Areca taro* is a plant of the genus taro in the family Araceae, and its edible part is the root bulb [1]. It is rich in various nutrients, such as carbohydrates, protein, and dietary fibre, and is low in fat. Also, it can enhance the immune function of the body and provide health benefits [2]. Since fresh *Areca taro* has a high moisture content and is prone to browning and spoilage, drying techniques are often utilised to extend the shelf life and improve the nutrient retention of the relative *Areca taro* products. Currently, industrially used taro drying techniques mainly include hot air drying [3], microwave drying [4], freeze drying [5,6], and infrared radiation drying [5]. In general, hot air drying has the disadvantages of low thermal efficiency and poor quality of dried products. Microwave drying has high thermal efficiency, but its drying uniformity is limited. Although freeze drying results in better-quality dried taro products, its disadvantages are high energy consumption and high equipment maintenance costs [6]. Moreover, infrared radiation drying technology has been widely used because food absorbs infrared radiation energy in the form of electromagnetic waves through molecular vibrations, thus resulting in rapid warming and a shortening of drying time [7]. However, it still has the drawbacks of low penetrability and uneven drying. Most of the existing taro drying methods still adopt the traditional single drying method, so there is an urgent need to develop a high-efficiency technology with low energy consumption and low cost, i.e., green and low-cost combined drying technology. This is the development trend of *Areca taro* drying.

To improve the drawback of uneven heat distribution in the process of drying taro by infrared radiation, spouted bed drying technology has attracted our attention. Spouted bed drying technology improves the traditional fluidized bed drying technology by using the material in the spraying bed for the fountain-type reciprocating movement, which contributes to the regular contact between the hot air and the materials, so the technology has a high heat transfer efficiency and drying uniformity [8,9,10]. In addition, infrared radiation drying can be easily combined with other drying technologies such as hot air drying and microwave drying, as well as spouted bed drying, i.e., infrared-assisted spouted bed drying (IRSBD) technology [11]. IRSBD has been successfully applied to drying food materials, and studies have shown that this combination can not only effectively improve the uniformity of infrared radiation drying but also solve the problem of the low thermal efficiency of traditional spouted bed drying, which has the advantages of a short drying time and low energy consumption [7,8]. For instance, IRSBD was applied to the drying of yam and peanut, respectively, with the advantages of energy-saving, low-consumption, and high-quality products [11,12]. However, to the best of our knowledge, the application of IRSBD for drying *Areca taro* has not been reported, and therefore the feasibility and relevant application parameters of IRSBD and the quality of the dried product are still unclear.

Based on the above, the aim of this study was to innovatively apply IRSBD technology for *Areca taro* drying and to investigate the effects of different parameters on its drying quality. Specifically, the effects of different drying temperatures and cutting sizes on the drying characteristics, temperature uniformity, and quality properties of the *Areca taro* were studied. Moreover, the drying process parameters of *Areca taro* were optimised with the objectives of energy savings and quality preservation to improve its commercial value. The obtained results can provide theoretical guidance for taro drying in practical production applications.

## 2. Materials and Methods

### 2.1. Sample Preparation

Fresh *Areca taro* was purchased from Hezhou, Guangxi Province, with an initial dry basis moisture content of (2.70 ± 0.2) g kg^−1^. *Areca taro* was peeled and cut into cubes with a cube cutter before the experiment.

### 2.2. Experimental Equipment

**Figure 1:**
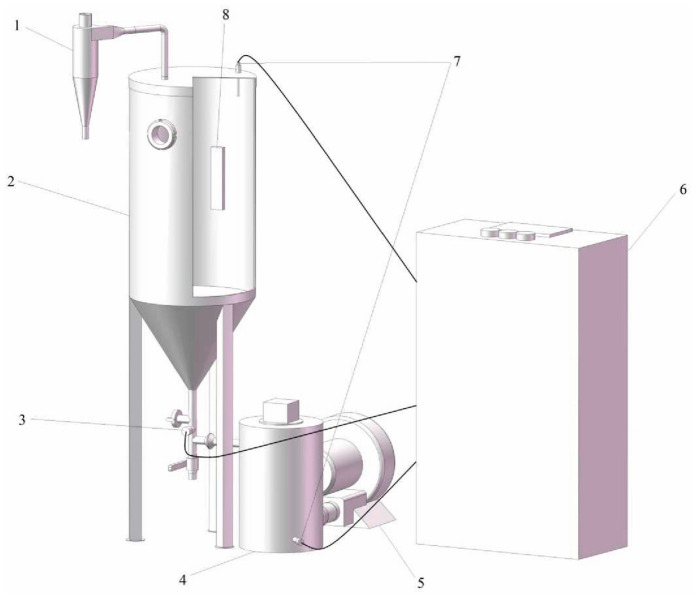
Structure diagram of the infrared-assisted spouted bed. 1: cyclone separator; 2: infrared-assisted spouted bed drying chamber; 3: wind speed sensor; 4: heating tank; 5: fans; 6: control cabinet; 7: temperature sensor; 8: infrared radiation plate.

As shown in Figure 1, the drying chamber of the infrared-assisted spouted bed consists of a cylinder with a top diameter of 350 mm and a height of 220 mm and a cone with a bottom diameter of 350 mm and a height of 235 mm. The drying chamber is equipped with an infrared heater for heating samples, and the material inside the infrared-assisted spouted bed is directed upwards by the airflow at the bottom. The material rises to a certain height and then falls back under gravity to form a fountain area. Because of the upward velocity of the air inside the instrument, an upward internal circulation is formed. To avoid heat loss, a circulating air design is used. The air temperature is detected by the thermal sensor, and the air speed is detected by the wind speed sensor.

### 2.3. Drying Experiments

When drying temperature was used as a variable, *Areca taro* with a size of 10 × 10 × 10 mm was dried at 45, 50, 55, and 60 °C, respectively. When cutting size was used as a variable, *Areca taro* was cut into cubes of 4 sizes (6 × 6 × 6 mm, 8 × 8 × 8 mm, 10 × 10 × 10 mm, and 12 × 12 × 12 mm) and dried at 50 °C. The reason we only discussed the samples with four different cutting sizes at 50 °C in this study was that the pre-experiment found that taro dried at 50 °C was of the best quality.

Since the weight of the samples decreased gradually during the drying process, a staged reduction of the air velocity was used to make the samples highly consistent during the spraying process. The results of the pre-test were analysed, and the air velocity was set at 6.5 m/s at the beginning of the experiment and decreased by 0.5 m/s every 10 min to a fixed velocity of 5 m/s until the end of the drying process. The weight of the samples was measured every 10 min. For each experimental group, 50 ± 0.5 g of *Areca taro* was placed in the infrared-assisted spouted bed, and drying was stopped when the moisture content of the samples fell below the safe moisture level.

### 2.4. Determination of Moisture Content on a Dry Basis

Fresh *Areca taro* was sliced to a thickness of 2–3 mm and dried in an oven (model 101 electrothermal constant temperature blast drying oven) at 105 °C. The mass of the sample before and after drying was recorded, and the moisture content of the fresh samples was calculated to be $\omega_{0}$. During the drying process, the weight and moisture content of the dried *Areca taro* samples ($m_{t}$) were measured every 10 min. The dry basis moisture content of the sample was calculated according to Equations (1) and (2) [13]:

(1) $$\omega_{t}=\frac{m_{t}-m_{0}(1-\omega_{0})}{m_{0}(1-\omega_{0})}\times 100\%$$

(2) $$\omega_{0}=\frac{m_{1}-m_{2}}{m_{1}}\times 100\%$$

where $\omega_{0}$, $m_{0}$, $m_{t}$, and $\omega_{t}$ represent the wet basis moisture content of the fresh sample (g/g), the weight of the fresh sample (g), the weight of the sample after drying at any time (g), and the dry basis moisture content of the sample after drying at any time (g/g), respectively. $m_{1}$ denotes the weight of the fresh sample before adiabatic drying (g), and $m_{2}$ denotes the weight of the sample after adiabatic drying (g).

### 2.5. Temperature Uniformity Evaluation

The uniformity of the temperature distribution of the samples at the end of drying was used to assess the drying uniformity. An infrared thermometer (FLIRI7, Shenzhen Yida Intelligent Technology Co., Ltd., Shenzhen, China) was used to determine the surface temperature of 10 randomly selected dried *Areca taro* samples. The centre of the sample surface was selected as the temperature measurement point, and the distance between the pyrometer gun and the sample was kept constant for each measurement. The coefficient of variation (CV) of temperature was used to evaluate the temperature uniformity. A smaller coefficient of variation correlates to better temperature uniformity.

### 2.6. Colour Analysis

The colour of dried *Areca taro* samples was analysed using a colorimeter (Color i 5D, X-rite Technology Co., Ltd., Grand Rapids, MI, USA), and fresh *Areca taro* samples were used as control. The chromatic aberration (∆*E*) was used to evaluate the colour parameters of the dried samples, and the colour difference was calculated using Equation (3):

(3) $$\Delta E=\sqrt{{\left(L^{*}-L_{0}^{*}\right)}^{2}+{\left(a^{*}-a_{0}^{*}\right)}^{2}+{\left(b^{*}-b_{0}^{*}\right)}^{2}}$$

where $L^{*}$, $a^{*}$ and $b^{*}$ represents brightness and darkness, redness and greenness, and yellowness and blueness of the dried *Areca taro*, respectively. $L_{0}^{*}$, $a_{0}^{*}$ and $b_{0}^{*}$ represent brightness and darkness, redness and greenness, and yellowness and blueness of the fresh *Areca taro*, respectively [12].

### 2.7. Determination of Rehydration Ratio

Referring to the method of Zhang et al. [14], 1 g (dry weight) of *Areca taro* was immersed in distilled water for the rehydration experiment. The samples were weighed after removing the surface water and placed at 25 °C. The mass of the samples was recorded every 20 min using an electronic balance until no further change was observed. The following Equation (4) was used to calculate the rehydration ratio (*RR*):

(4) $$RR=\frac{m_{t}}{m_{0}}$$

where $m_{t}$ is the sample mass (g) after rehydration, and $m_{0}$ is the initial mass (g) of the dried sample.

### 2.8. Determination of Total Phenol Content (TPC)

The dried *Areca taro* was first powdered using a pulveriser (OX-C949 Multifunctional Pulverizer, Wuyi Haina Electrical Appliance Co., Ltd., Wuyi, China). Then, the obtained powder (0.8 g) was extracted with 8 mL of 80% ethanol (Tianjin Deen Chemical Ragent Co., Ltd, Tianjin, China) in a ultrasonic equipment (KQ-500DE CNC ultrasonic equipment, Kunshan Ultrasonic Instrument Co., Ltd., Kunshan, China) for one hour. The mixture was centrifuged at 5000 r/min for 10 min, and the supernatant was collected for the determination of TPC, total flavonoid content, and antioxidant activity. For TPC determination, we adopted the Folin–Ciocâlteu colorimetric method, which is the most commonly used [15]. Specifically, the standard curve was plotted using gallic acid as the standard, and the regression equation was Y = 14.339X + 0.0037, *R*^2^ = 0.9994. Initially, 0.2 mL of Folin–Ciocâlteu solution (Tianjin Deen Chemical Ragent Co., Ltd., Tianjin, China) was added to 0.1 mL of taro ethanol extract. The solution was allowed to stand for 5 min and then mixed with 0.6 mL Na_2_CO_3_ (75 g L^−1^). The total volume of this solution was made up to 2 mL with the addition of ethanol. The mixture was allowed to stand for 1 h in the dark and then centrifuged at 5000 r/min for 10 min at 25 °C. The supernatant was collected, and the absorbance at 760 nm was measured by a multifunctional enzyme labeller (SPARK-type multifunctional enzyme labeller, Tecan Austria GmbH, Groedig, Austria). The TPC was calculated from the standard curve. The results were expressed as mg gallic acid equivalents (GAE) per g of dry matter (DM) (mg GAE g^−1^ DM) [16].

### 2.9. Determination of Total Flavonoid Content (TFC)

The total flavonoid content was determined by the spectrophotometric method [17]. The supernatant obtained in 2.9 was added to a tube, followed by 0.2 mL of Na_2_NO_2_ and 0.2 mL of 10% Al(NO_3_)_3_. The mixture was allowed to stand for 6 min, and then 2 mL of 4% NaOH was added. The solution was thoroughly mixed and allowed to stand for 15 min in the dark. Then, the absorbance at 510 nm was read by a multifunctional enzyme labeller (SPARK-type multifunctional enzyme labeller, Tecan Austria GmbH, Groedig, Austria). The standard curve was plotted using rutin as the standard, and the regression equation was Y = 2.3287X − 0.0031, R^2^ = 0.9989. The TFC was calculated from the standard curve and expressed as mg rutin equivalent (RE) per g of DM (mg RE g^−1^ DM) [12].

### 2.10. Determination of Antioxidant Activity

The antioxidant activity of the sample was evaluated using 1,1-diphenyl-2-picrylhydrazyl (DPPH) (Shanghai Yuanye Bio-Technology Co., Ltd., Shanghai, China) radical scavenging capacity and 2,20-azino-bis (3-ethylbenzothiazoline-6-sulfonic acid) (ABTS) (Shanghai Yuanye Bio-Technology Co., Ltd., Shanghai, China) radical scavenging capacity [18,19]. For DPPH determination, 0.1 of the mL supernatant obtained in *2.9* was mixed with 0.9 mL of 80% ethanol and 1 mL of DPPH solution. After the sample was kept at room temperature for 30 min in the dark, the absorbance was measured at 517 nm using a multifunctional microplate reader, and the sample concentration was calculated via the standard curve (Y = 1.0424 − 61.05X, R^2^ = 0.9935). The results were expressed as mg of vitamin C (Vc) equivalent per g of DM (mg Vc g^−1^ DM) [12]. For ABTS determination, 0.1 mL of supernatant was mixed with 4.9 mL of 80% ethanol. Then, 0.1 mL of the solution was mixed with 1.9 mL of 80% ethanol. After the sample was kept at room temperature for 15 min in the dark, the absorbance was measured at 734 nm using a multifunctional enzyme labeller (SPARK-type multifunctional enzyme labeller, Tecan Austria GmbH, Groedig, Austria).The sample concentration was calculated via the standard curve (Y = 0.544 − 28.7X, R^2^ = 0.0002). The results were expressed as mg of vitamin C (Vc) equivalent per g of DM (mg Vc g^−1^ DM) [12].

### 2.11. Determination of the Total Energy Consumption

The difference between the metre readings of the infrared-assisted spouted bed at the end of drying and at the beginning of drying was used as the total energy consumption (kW·h) for the drying process.

### 2.12. Statistical Analysis

All the experiments were repeated three times in parallel. Plotting was performed using Origin version 2021 software (OriginLab Corp., Northampton, MA, USA). One-way analysis of variance (ANOVA) and Duncan’s multiple comparison test with 95% confidence (*p* < 0.05) were performed using SPSS 24.0 (SPSS Inc., Chicago, IL, USA) to analyse significant differences between groups.

## 3. Results

### 3.1. Drying Characteristics

**Figure 2:**
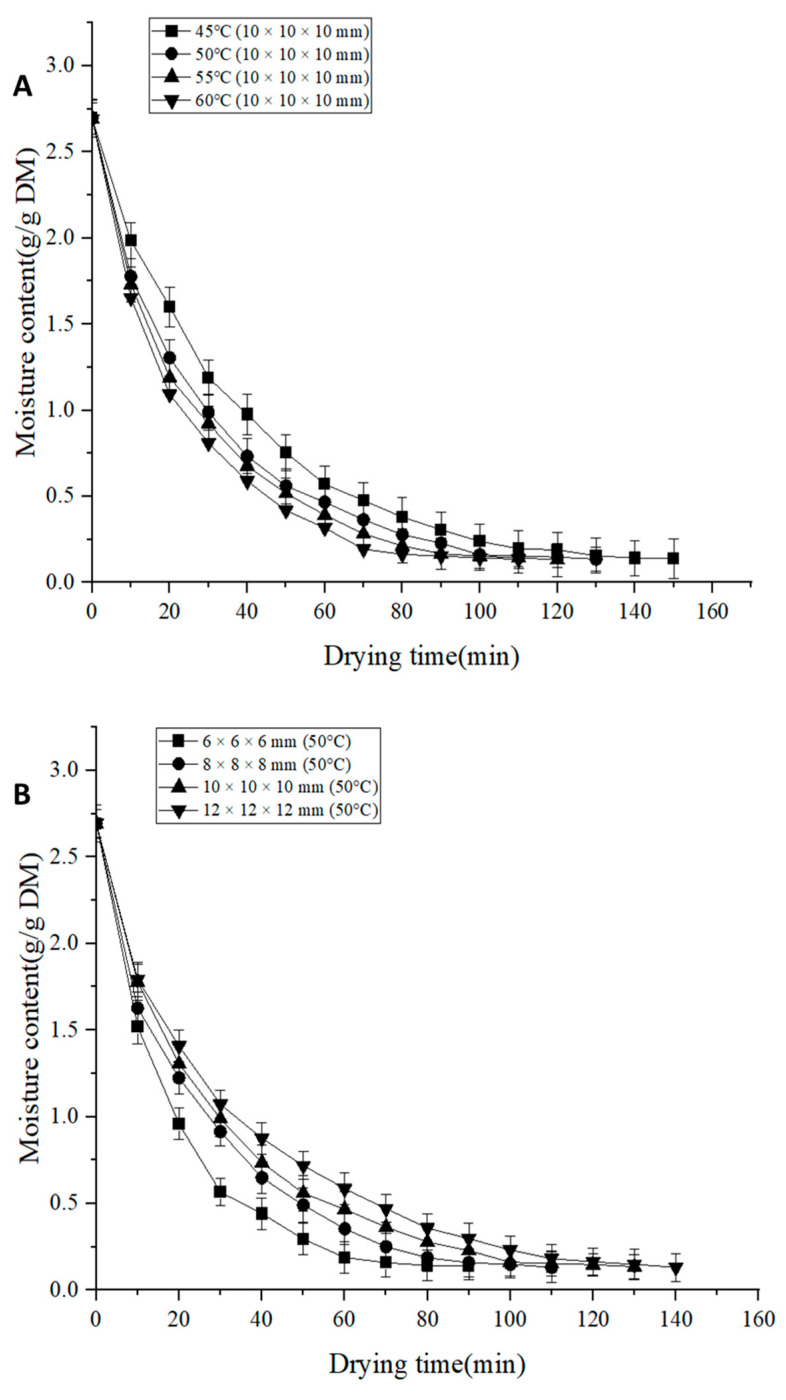
Drying characteristics under different drying conditions. (**A**): drying curves of *Areca taro* samples at different drying temperatures; (**B**): drying curves of *Areca taro* samples at different cutting sizes.

As shown in Figure 2A, the increase in drying temperature resulted in a shorter drying time, and a decreasing trend in the dry basis moisture content of *Areca taro* was observed during the drying process. Meanwhile, *Areca taro* had the longest drying time, about 150 min at 45 °C. The drying temperatures of 50, 55, and 60 °C were shorter by 7.14%, 21.4%, and 28.6%, respectively, compared to 45 °C. Therefore, increasing the drying temperature of IRSBD can significantly reduce the drying time, improve the drying efficiency, and speed up the drying rate. This is because a higher temperature can increase the temperature difference between the heat transfer medium and the sample, thus increasing the heat transfer rate and moisture evaporation rate of the sample. In addition, at a certain air humidity, the relative saturation humidity of the air decreases as the temperature increases, and the driving force for the moisture diffusion from the sample surface increases. High-temperature hot air provides a higher external heat flux and promotes moisture diffusion, thus increasing the drying rate and shortening the drying time [12].

Figure 2B shows the variation in dry basis moisture content of *Areca taro* samples under four cutting sizes for different drying times at 50 °C. It can be seen that reducing the cutting size shortens the drying time, i.e., the shortest drying time was required for *Areca taro* with a cutting size of 6 × 6 × 6 mm. The drying time of taro in this group was shortened by 18.18%, 30.77%, and 35.71% compared to the experimental groups with cutting sizes of 8 × 8 × 8 mm, 10 × 10 × 10 mm, and 12 × 12 × 12 mm, respectively. Previous studies have shown that the smaller the cutting size of the sample, the shorter the distance of moisture transfer from the interior to the surface and the shorter the drying time [20,21]. In addition, a smaller cutting size results in less resistance to moisture transfer and a faster evaporation rate [22]. Conversely, a larger cutting size increases the pathway for moisture to diffuse out of the taro, reducing the evaporation rate [23]. Due to the increase in heat transfer resistance, the drying rate decreased with the increase in cutting size, and therefore the drying time was prolonged. The above results are in agreement with the findings of Sadeghi et al. [22].

### 3.2. Drying Uniformity

**Figure 3:**
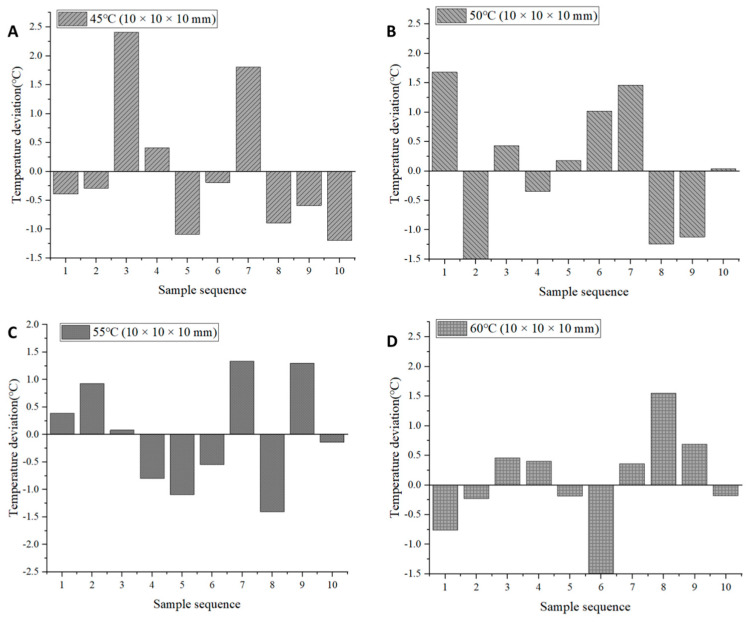
Temperature deviation distribution of ten randomly selected dried *Areca taro* under different drying temperatures. (**A**): 45 °C (10 × 10 × 10 mm); (**B**): 50 °C (10 × 10 × 10 mm); (**C**): 55 °C (10 × 10 × 10 mm); (**D**): 60 °C (10 × 10 × 10 mm).

**Figure 4:**
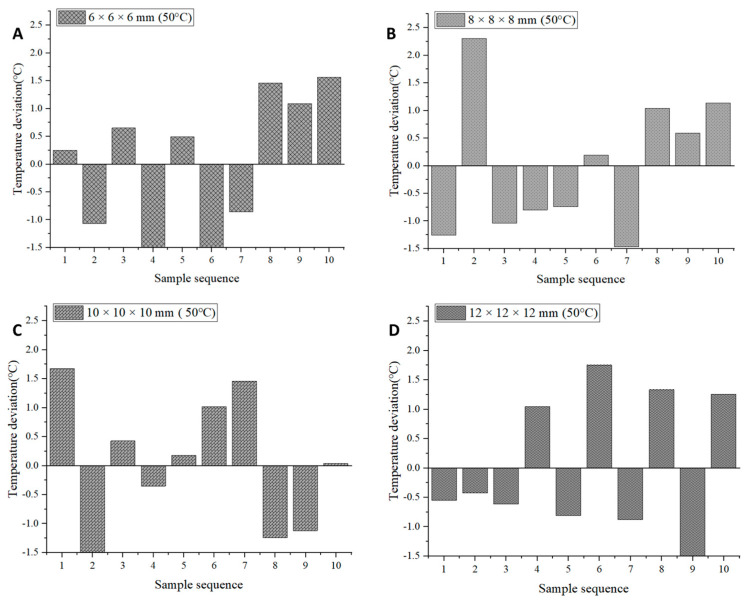
Temperature deviation distribution of ten randomly selected dried *Areca taro* under different cutting sizes. (**A**): 6 × 6 × 6 mm (50 °C); (**B**): 8 × 8 × 8 mm (50 °C); (**C**): 10 × 10 × 10 mm (50 °C); (**D**): 12 × 12 × 12 mm (50 °C).

Temperature uniformity is one of the indicators for evaluating the uniformity of the drying process of *Areca taro*. Inhomogeneous drying can cause excessive drying of *Areca taro* and make it difficult to reach a safe moisture content, thus affecting its quality and storage. Since the infrared heat source scatters electromagnetic waves onto the surface of *Areca taro* from all directions, inconsistencies in the radiation intensity as well as changes in the relative position between the *Areca taro* blocks and the heat source can affect the temperature uniformity of the IRSBD process [24]. In this study, the temperature distribution of *Areca taro* blocks at the end of drying was measured to assess the uniformity of drying. Figure 3 and Figure 4 and Table 1 show the results of the temperature of 10 randomly selected dried *Areca taro* samples under different drying conditions. As can be seen from Table 1, when the drying temperature was taken as the variable, the CV value decreased gradually with the increasing drying temperature, i.e., the CV value was the smallest at 60 °C, and the difference between the average surface temperature and the drying temperature of *Areca taro* was the smallest. Hence, the drying uniformity of *Areca taro* was optimal at 60 °C. This phenomenon can be explained by the fact that, firstly, the infrared radiation has internal heating properties, so that the internal temperature of the sample is higher than its surface temperature. Secondly, the hot air in the infrared-assisted spouted bed balances the surface temperature of the sample with the temperature of the hot air, i.e., the temperature of the sample is affected by both convection and thermal radiation. Specifically, the balance of thermal convection and thermal radiation heating of IRSBD at higher temperatures can reduce the temperature difference between the dried *Areca taro* blocks at the end of drying and improve the drying uniformity [12].

Moreover, at 50 °C, when the cutting size of the *Areca taro* was selected as a variable, the CV value tended to decrease and then increase with increasing cutting size. The CV was the lowest when the cutting size of the *Areca taro* was 10 × 10 × 10 mm, and the difference between the average surface temperature of the *Areca taro* blocks and the drying temperature was the smallest, indicating that the drying uniformity was optimal when the cutting size was 10 × 10 × 10 mm. Reducing the cutting size of the *Areca taro* could aggravate surface hardening, resulting in poor drying uniformity. When the cutting size was increased from 10 × 10 × 10 mm to 12 × 12 × 12 mm, infrared radiation had difficulty reaching the inside of the *Areca taro* blocks due to the long migration path of moisture, which caused a slower increase in the internal temperature and affected the drying uniformity.

### 3.3. Colour Analysis

Colour is an important indicator for evaluating dried *Areca taro* products and affects their commercial value and consumer acceptance. Lower ∆*E* and b* values and higher L* values indicate the better colour of the dried *Areca taro*. Table 2 shows the colour parameters of the dried *Areca taro*, and it can be seen that the L* values of the dried samples were significantly higher than those of the fresh samples (*p* < 0.05). When drying temperature was selected as the variable, IRSBD had no significant effect on the L* values of *Areca taro* (*p* > 0.05). In addition, the b* and ∆*E* values of the *Areca taro* were the lowest at 50 °C. Generally, colour changes are caused by enzymatic browning, non-enzymatic browning, and degradation of the pigments during drying. Drying of *Areca taro* at 45 °C for a longer period of time resulted in prolonged enzymatic browning and oxidative reactions in *Areca taro* samples. While the drying temperature was gradually increased from 50 °C, the higher drying temperature caused the formation of hot spots inside the taro pieces, which promoted the Maillard reaction and the caramelization reaction [12]. Moreover, the rate of browning due to the Maillard reaction increased 2 to 5 times for every 10 °C increase in temperature [25]. As a result, the colour of the samples became darker, and the values of ∆*E* and b* increased.

When the cutting size of the samples was increased from 6 × 6 × 6 mm to 10 × 10 × 10 mm, the ∆*E* and b* values decreased. When the cutting size was increased from 10 × 10 × 10 mm to 12 × 12 × 12 mm, the ∆*E* and b* values increased, and both values reached a minimum at a cutting size of 10 × 10 × 10 mm. This is because the smaller cutting size can severely damage the internal tissues and cells of *Areca taro* during drying, leading to enzymatic browning [26]. Next, when the cutting size was increased from 10 × 10 × 10 mm to 12 × 12 × 12 mm, the larger cutting size resulted in a longer drying time, which increased the oxidative reaction time of *Areca taro*. Additionally, the larger size of the cut pieces increased the amount of internal pigment deposition in the dried *Areca taro*. Therefore, a drying temperature of 50 °C and a cutting size of 10 × 10 × 10 mm yielded the best colour of the dried *Areca taro*. Notably, compared with the microwave-dried *Areca taro* reported by Wei et al. [3], the samples dried by IRSBD in this study had a better L* value, ∆*E* value, shorter drying time, lower energy consumption, as well as better colour.

### 3.4. Rehydration Ratio

**Figure 5:**
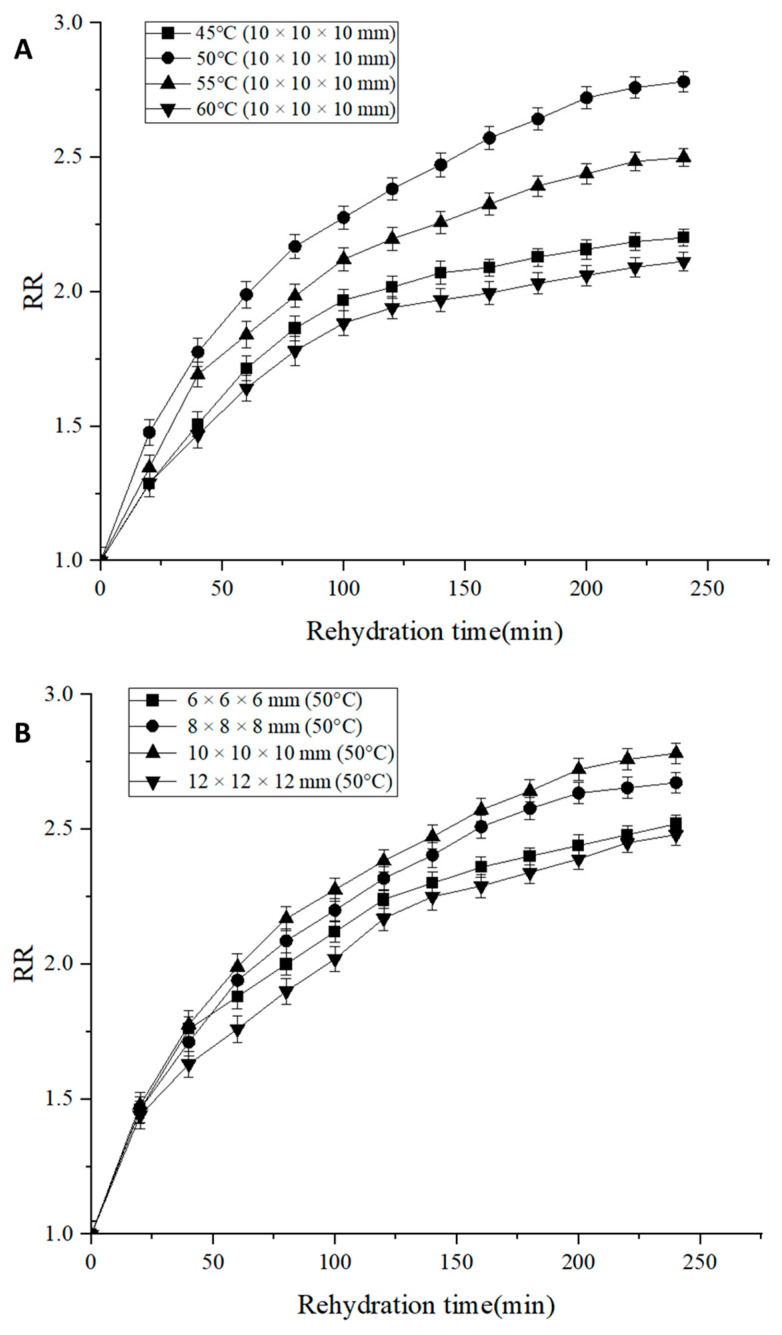
*RR* of the dried *Areca taro* under various drying conditions. (**A**): *RR* of *Areca taro* samples at different drying temperatures; (**B**): *RR* of *Areca taro* samples with different cutting sizes.

Drying causes irreversible changes in the composition and structure of the dried products, thus limiting the recovery of their shape and size. *RR* can be used to measure the destructive effects of the drying process on *Areca taro*. Figure 5 shows the variation of *RR* values of dried *Areca taro* under different conditions. From Figure 5A, it can be seen that with the increase in drying temperature, the *RR* values showed a trend of increasing and then decreasing. Specifically, when the drying temperature was 50 °C, the *RR* values reached their maximum, indicating that the structure of dried *Areca taro* was damaged to a lesser extent under this condition. When the drying temperature was 45 °C, the samples were observed to be heavily wrinkled and tightly structured due to the long drying time, which was not conducive to the absorption of water during rehydration. When the drying temperature was increased from 50 °C to 60 °C, the difference in vapour pressure between the surface and the interior of the dried *Areca taro* increased, leading to the hardening and crumple of the taro surface. Meanwhile, the high level of internal destruction of *Areca taro* during the drying process reduced the permeability of the cell wall and was not conducive to rehydration [26].

As can be seen from Figure 5B, at 50 °C, the rehydration ratio increased when the cutting size was increased from 6 × 6 × 6 mm to 10 × 10 × 10 mm and decreased as the cutting size continued to increase. The increased *RR* values with increasing cutting sizes were probably due to the increase in surface area of the samples, which benefited their rehydration. But when the cutting size was further increased from 10 × 10 × 10 mm to 12 × 12 × 12 mm, the increase in drying time led to an increase in the degree of disruption of *Areca taro* microstructure, which reduced the water holding capacity of the samples and decreased the *RR* value [26,27]. Based on the above, the maximum *RR* value reached 2.78 when the cutting size was 10 × 10 × 10 mm and the drying temperature was 50 °C.

### 3.5. Bioactive Compounds

Polyphenols are important nutrients in *Areca taro* that are easily lost due to temperature and oxidation in food processing. Flavonoids are functional substances with multiple effects that are widely found in plants and are susceptible to oxidative degradation during drying [28]. Table 3 shows that as the drying temperature was increased from 45 °C to 50 °C, both TPC and TFC increased and reached their maximum values at 50 °C. Then, both TPC and TFC decreased as the temperature increased from 50 °C to 60 °C. This phenomenon can be explained for the following reasons: Firstly, the longer drying time and the higher polyphenol oxidase activity at 45 °C resulted in polyphenols being more easily oxidised and enzymatically cleaved. As the drying temperature increased from 45 °C to 50 °C, the activity of polyphenol oxidase decreased and the drying time was shortened, resulting in a shorter contact time between phenolic substances and air, which caused the increase of TPC and TFC in samples. Secondly, higher temperatures also led to fragmentation of plant cells, reduction of cell membrane permeability, and rupture of the covalent bonds, which promoted the release of TPC and TFC. The drying temperature of 50 °C could promote the oxidation of hydroxyl radicals, which in turn produced more metabolic TPC and TFC [12]. Finally, the structure of polyphenols and flavonoids was destroyed when the temperature increased from 50 °C to 60 °C. As a result, the degradation rate of these two substances increased and their content decreased.

In addition, both TPC and TFC contents were highest at 50 °C when the cutting size was 10 × 10 × 10 mm. When the cutting size increased from 6 × 6 × 6 mm to 10 × 10 × 10 mm, the drying time was prolonged, and the heat treatment broke more molecular bonds, releasing more TPC and TFC [29]. The TPC and TFC content decreased when the cutting size was increased from 10 × 10 × 10 mm to 12 × 12 × 12 mm. This was caused by the increase in drying time and the prolonged degradation of TPC and TFC. Michalczyk et al. [30] also demonstrated that long periods of high-temperature drying resulted in a significant decrease in phenolic content. Compared to other drying methods, IRSBD is more favourable for the retention of polyphenols and flavonoids. Infrared radiation is converted into energy through molecular vibration, which is rapidly absorbed into the centre of the material during the drying process [31]. The covalent bonds of bound polyphenols were broken by infrared radiation, which further contributed to the release of small-molecule phenolic compounds [31].

### 3.6. Antioxidant Activity

DPPH and ABTS scavenging capacity were used to determine the antioxidant activity of *Areca taro*. As shown in Table 3, there was a significant difference (*p* < 0.05) in the free radical scavenging capacities of the *Areca taro* samples dried at different temperatures. Specifically, both DPPH and ABTS values increased when the temperature was increased from 45 °C to 50 °C and decreased when the temperature was increased from 50 °C to 60 °C. Therefore, *Areca taro* dried at 50 °C had the maximum DPPH and ABTS radical scavenging capacity, i.e., the maximum antioxidant activity. Based on this, the antioxidant activity tests were further carried out under four different cutting sizes that dried at 50 °C.

At 50 °C, the ABTS radical scavenging capacity increased and then decreased when the cutting size was increased from 6 × 6 × 6 mm to 12 × 12 × 12 mm and reached the maximum value at 10 × 10 × 10 mm. The DPPH radical scavenging capacity of *Areca taro* was similar to the ABTS radical scavenging capacity of *Areca taro*. Also, this trend corresponds to the content of TPC and TFC, i.e., the higher the content of TPC and TFC, the higher the antioxidant capacity of *Areca taro*. Previous studies have shown that polyphenols and flavonoids have the ability to scavenge reactive oxygen species and benefit the free radical scavenging capacity of dehydrated foods [32,33,34]. In summary, when the drying temperature and cutting size variables were analysed together, the maximum radical scavenging capacity was reached at 50 °C with a cutting size of 10 × 10 × 10 mm.

### 3.7. Energy Consumption

**Figure 6:**
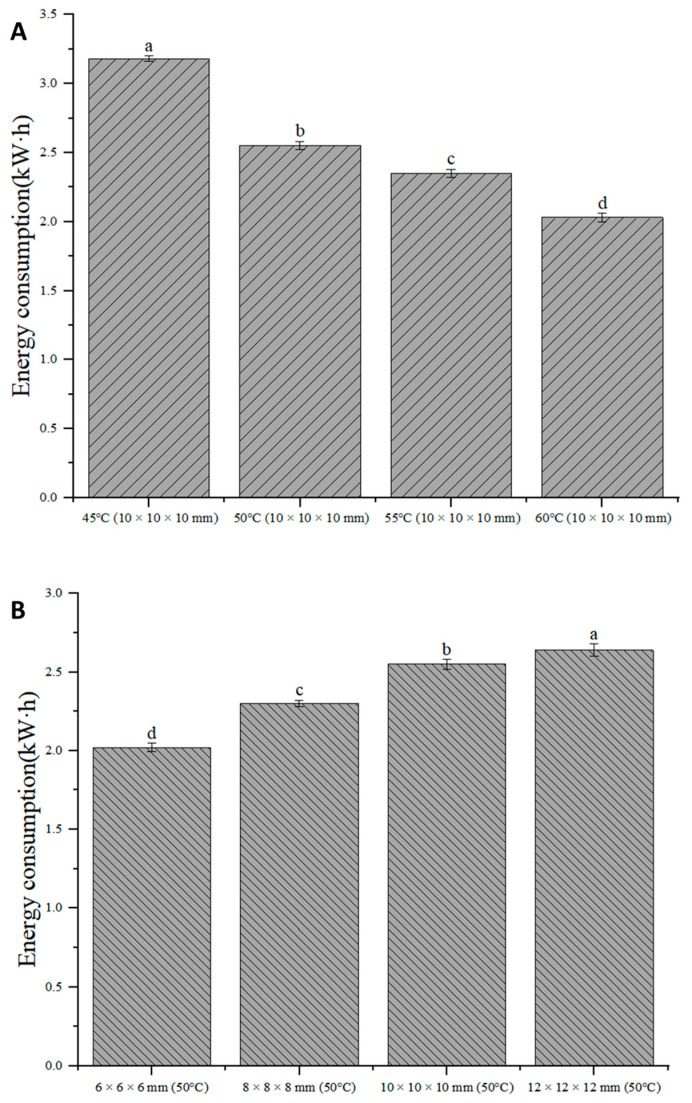
Energy consumption of IRSBD under different drying conditions. (**A**): energy consumption of *Areca taro* samples at different drying temperatures; (**B**): energy consumption of *Areca taro* samples at different cutting sizes. Different letters in the same column indicate a significant difference (*p* < 0.05).

Energy consumption is one of the important indices for evaluating the efficiency of drying methods. The energy consumption of IRSBD is shown in Figure 6. When the drying temperature was used as the variable, the drying energy consumption in descending order was as follows: 45 °C (3.18 kW·h) > 50 °C (2.55 kW·h) > 55 °C (2.35 kW·h) > 60 °C (2.03 kW·h). Compared to the IRSBD at 45°C, the drying energy consumption at 50 °C, 55 °C, and 60 °C decreased by 20%, 26%, and 36%, respectively. This result shows that a higher temperature resulted in a shorter drying time and lower energy consumption. This is because the internal moisture migration resistance of the same material is the same, and the drying rate is related to the moisture migration capacity and the surface gasification ability. Thus, the increase in drying temperature led to an increase in moisture migration and gasification capacity, which in turn improved drying efficiency and reduced drying energy consumption [35].

In addition, the drying energy consumption at 50 °C in descending order with the cutting size as the variable was 12 × 12 × 12 mm (2.64 kW·h) > 10 × 10 × 10 mm (2.55 kW·h) > 8 × 8 × 8 mm (2.3 kW·h) > 6 × 6 × 6 mm (2.02 kW·h). This result indicates that reducing the cutting size can reduce the resistance to heat and mass transfer, shorten the drying time, and reduce drying energy consumption. The energy consumption of IRSBD at different temperatures and cutting sizes was significantly different (*p* < 0.05). Therefore, under the premise of ensuring product quality, it is suggested that the drying temperature be increased and the cutting size be reduced.

## 4. Conclusions

IRSBD technology integrates the advantages of infrared radiation drying and spraying bed drying, which have good drying uniformity and heat transfer efficiency. Therefore, the effects of different drying temperatures and cutting sizes on the drying characteristics, temperature uniformity, and quality properties of the *Areca taro* dried by IRSBD were evaluated. Important findings include: (1) That increasing the drying temperature and decreasing the cutting size were conducive to shortening the drying time and reducing the drying energy consumption. (2) Increasing the temperature improved the drying uniformity. (3) The optimal *Areca taro* samples with high quality were produced at a cutting size of 10 × 10 × 10 mm and a drying temperature of 50 °C, and the process could be improved by reducing the drying energy consumption. This study provides a theoretical basis for the optimisation of drying technology applied to *Areca taro*.

To expand the application of infrared-assisted spouted bed-dried *Areca taro* in the development of leisure food, future plans include investigating the effects of different drying temperatures and cutting sizes on the texture characteristics (e.g., hardness and crispness), shrinkage ratio, and microstructure of the *Areca taro* dried by IRSBD.

## Figures and Tables

**Table 1:** Temperature parameters of ten random *Areca taro* samples at the end of drying.

Samples	Average Temperature (°C)	CV (%)	Average Temperature (°C)−Set Temperature (°C)
45 °C (10 × 10 × 10 mm)	45.49	2.67	0.49
50 °C (10 × 10 × 10 mm)	50.38	2.47	0.38
55 °C (10 × 10 × 10 mm)	54.71	1.81	−0.29
60 °C (10 × 10 × 10 mm)	59.88	1.65	−0.12
6 × 6 × 6 mm (50 °C)	49.55	2.62	−0.46
8 × 8 × 8 mm (50 °C)	50.44	2.48	0.44
12 × 12 × 12 mm (50 °C)	50.72	2.52	0.72

**Table 2:** Colour parameters of the dried *Areca taro*.

Samples	L* Value	a* Value	b* Value	∆*E*
Fresh samples	84.96 ± 1.57 ^c^	0.91 ± 1.17 ^ab^	6.52 ± 0.09 ^e^	
45 °C (10 × 10 × 10 mm)	86.89 ± 0.03 ^b^	1.89 ± 0.04 ^a^	10.99 ± 0.05 ^b^	3.82 ± 0.03 ^cd^
50 °C (10 × 10 × 10 mm)	87.59 ± 0.03 ^b^	1.58 ± 0.05 ^ab^	8.2 ± 0.69 ^d^	2.83 ± 0.05 ^e^
55 °C (10 × 10 × 10 mm)	87.42 ± 0.05 ^b^	1.48 ± 0.04 ^ab^	10.43 ± 0.04 ^b^	2.99 ± 0.03 ^de^
60 °C (10 × 10 × 10 mm)	86.98 ± 0.03 ^b^	1.38 ± 0.03 ^ab^	12.02 ± 0.04 ^a^	4.76 ± 0.04 ^bc^
6 × 6 × 6 mm (50 °C)	90.89 ± 0.44 ^a^	1.01 ± 0.07 ^b^	8.74 ± 0.05 ^cd^	6.08 ± 0.44 ^a^
8 × 8 × 8 mm (50 °C)	89.89 ± 1.03 ^a^	1.16 ± 0.23 ^ab^	8.72 ± 0.59 ^cd^	5 ± 1.01 ^b^
12 × 12 × 12 mm (50 °C)	89.97 ± 0.55 ^a^	0.91 ± 0.05 ^b^	9.23 ± 0.17 ^c^	5.28 ± 0.58 ^ab^

Different letters in the same column indicate a significant difference (*p* < 0.05).

**Table 3:** TPC, TFC, and antioxidant activity of dried *Areca taro*.

Samples	TPC (mg GAE/g DM)	TFC (mg RE/g DM)	DPPH Scavenging Capacity (mg Vc/g DM)	ABTS Scavenging Capacity (mg Vc/g DM)
45 °C (10 × 10 × 10 mm)	0.39 ± 0.04 ^ab^	1.87 ± 0.23 ^b^	1.54 ± 0.01 ^ab^	6.7 ± 0.02 ^a^
50 °C (10 × 10 × 10 mm)	0.43 ± 0.01 ^a^	2.5 ± 0.01 ^a^	1.55 ± 0.01 ^a^	7 ± 0.18 ^a^
55 °C (10 × 10 × 10 mm)	0.25 ± 0.01 ^ef^	2.02 ± 0.01 ^b^	1.47 ± 0.02 ^c^	5.54 ± 0.01 ^b^
60 °C (10 × 10 × 10 mm)	0.22 ± 0.01 ^f^	1.29 ± 0.03 ^d^	1.43 ± 0.03 ^d^	4.61 ± 0.95 ^c^
6 × 6 × 6 mm (50 °C)	0.32 ± 0.47 ^cd^	1.19 ± 0.02 ^d^	1.49 ± 0.01 ^c^	3.4 ± 0.10 ^d^
8 × 8 × 8 mm (50 °C)	0.38 ± 0.02 ^bc^	1.51 ± 0.05 ^c^	1.52 ± 0.01 ^b^	4.64 ± 0.02 ^c^
12 × 12 × 12 mm (50 °C)	0.29 ± 0.01 ^de^	1.5 ± 0.12 ^c^	1.54 ± 0.01 ^ab^	4.82 ± 0.28 ^bc^

Different letters in the same column indicate a significant difference (*p* < 0.05).

## Data Availability

The data presented in this study are available on request from the corresponding author. The data are not publicly available because research is ongoing.
